# Supplementary material for: A comparative analysis of factors associated with modern contraceptive use among youth in Northern and Southern Nigeria: A cross-sectional population-based survey (2011–2021)
Source: PLOS Glob Public Health. 2025 Jun 2;5(6):e0004685. doi: 10.1371/journal.pgph.0004685 (PMC12129170; doi:10.1371/journal.pgph.0004685)
Supplement: Attachment — Submitted filename: Rebuttal response.docx [file pgph.0004685.s004.docx]

**Rebuttal Response**

| **No** | **Comment** | **Response to Reviewer** |
| --- | --- | --- |
| **Introduction** | | |
| 1 | Introduction has touched multiple angles. Although: The low contraceptive prevalence rate for Africa and Sub-Saharan Africa need to be clarified. (i.e. in Africa, the CPR remains much lower, particularly in Sub-Saharan Africa, where only about 25% of women aged 15-49 use modern contraceptive methods. What is the cut-off point?). | Thank you for your comment. I have revised the introduction to clarify the global contraceptive prevalence rate and to provide a clear comparison of modern contraceptive use among women aged 15–19 and 20–24 years across different regions. |
| **Methodology** | | |
| 2 | Methods: The methods used, including the study design, area, sample, sampling technique, instrument, and analysis, were valid and reliable, which led to the final results. All comments were addressed well, except for comment number 02. How was the sample size determined? Was a formula used? Please provide it. | Thank you for your comment. I have clarified that a specific formula was not used, as the study relied on **MICS data from 2011–2021**, selecting all women aged **15–24 years** who met the inclusion criteria. This aligns with similar studies using **MICS and other large-scale demographic databases**, where sample selection follows **predefined survey methodologies** rather than independent calculations. |
